# Caveolin-1, GATA-3, and Ki67 expressions and their correlation with pathological findings in canine bladder urothelial carcinoma

**DOI:** 10.3389/fvets.2022.986269

**Published:** 2022-10-10

**Authors:** Verônica Mollica Govoni, Claudio Pigoli, Eleonora Brambilla, Felipe Augusto Ruiz Sueiro, Rafael Torres Neto, Renee Laufer-Amorim, Juliany Gomes Quitzan, Valeria Grieco, Carlos Eduardo Fonseca-Alves

**Affiliations:** ^1^Department of Veterinary Surgery and Animal Reproduction, School of Veterinary Medicine and Animal Science, São Paulo State University – UNESP, Botucatu, Brazil; ^2^Laboratorio di Istologia, Sede Territoriale di Milano, Istituto Zooprofilattico Sperimentale della Lombardia e dell'Emilia Romagna (IZSLER), Milan, Italy; ^3^Department of Veterinary Medicine and Animal Sciences, Università degli Studi di Milano, Milan, Italy; ^4^VetPat Laboratory, Campinas, Brazil; ^5^VetMol Laboratory, Botucatu, Brazil; ^6^Department of Veterinary Clinic, School of Veterinary Medicine and Animal Science, São Paulo State University – UNESP, Botucatu, Brazil; ^7^Institute of Health Sciences, Paulista University – UNIP, Bauru, Brazil

**Keywords:** cancer, dog, biomarker, immunohistochemistry, neoplasia

## Abstract

The bladder urothelial carcinoma (UC) represents ~2% of malignant neoplasms in dogs and is a therapeutic challenge in veterinary medicine. Although it is considered the most common bladder cancer in dogs, few previous studies have investigated different markers that correlate with clinical and pathological parameters. Therefore, this study aimed to evaluate Caveolin-1, GATA-3, and Ki67 immunostaining in canine UC samples to evaluate their correlations with histopathological variables. Thirty tumor samples were obtained, and Caveolin-1, GATA-3, and Ki67 expression was assessed by immunohistochemistry and associated with pathological factors by univariate and multivariate analyses. Among the histopathological findings, lymphatic invasion was identified in 53.33% of the tumors, and the mean mitotic count (MC) was 31.82 ± 26.26. Caveolin-1 showed mild-to-high cytoplasmic expression in neoplastic cells, whereas GATA-3 showed mild-to-high nuclear expression. The Ki67 expression revealed a mean of 24.14 ± 16.88% positive cells. In the univariate analysis, no association was found between each marker and the pathological findings. On the other hand, in multivariate analysis, we identified a positive correlation between GATA-3 and MC and a negative correlation between Caveolin-1 and MC. Moreover, lymphatic invasion was positively correlated with histological type and grade, and negatively correlated with MC. In addition, the histological type was positively correlated with the histological grade. Overall, our results indicate that Caveolin-1 and GATA-3 expression could be promising markers for bladder UC aggressiveness.

## Introduction

Urothelial carcinoma (UC) represents 1.5–2% of naturally-occurring cancers in dogs, and due to the similarities between the dog and human diseases, the canine muscle-invasive bladder tumor has been used as a study model for the human disease (1–4). Although invasive bladder UC is lethal in 50% of cases in humans (1), ~70% of patients have a non-invasive bladder tumor (5, 6). In contrast, the disease in dogs is mainly represented by the invasive form (4), and the majority of canine UC is located in the vesical trigone (1, 4). Due to these factors, the treatment of bladder tumors in dogs is challenging, and most patients are treated with chemotherapy.

Gambim et al. (7) performed a meta-analysis of literature on biomarkers for canine UC and demonstrated a lack of markers for this tumor subtype. Previous studies that investigated biomarkers have usually described the marker expression pattern with no association with clinical and pathological factors (8). Therefore, little is known about factors associated with UC aggressiveness, progression, and invasion (7). Gambim et al. (7) also found previous studies with transcriptomic data and performed *in silico* analysis to identify potential biomarkers for canine UC. Among these biomarkers, Caveolin-1 and GATA-binding protein 3 (GATA-3) were identified as promising markers of tumor aggressiveness.

Caveolins are plasma membrane proteins that regulate complex intracellular signaling pathways related to cancer progression (9). Caveolin-1 is an important signaling protein that has been associated with several cancers, and its overexpression is associated with cancer progression and resistance to therapies (9–12). Caveolin-1 expression has also been investigated for human UC, both in the bladder and the upper urinary tract, and correlated with clinicopathological factors and cancer progression (9, 10). This protein is involved in several cellular biological processes such as endocytosis, vesicular transport, and signaling pathways (11, 12). In veterinary medicine, no studies have investigated the predictive value or the possible association between Caveolin-1 expression and clinicopathological variables in canine bladder UC.

GATA-3 is a zinc finger transcription factor that is mainly involved in the differentiation and cell specification processes of tissues such as the urothelium and breast epithelium (13, 14). It has been studied as a marker for human UC, both related to diagnosis and clinicopathological factors, such as histological grade, histological type, and staging, to investigate its role as a predictor of the behavior of this tumor type (15–17). Although it is one of the most important markers for urothelial differentiation, to the best of our knowledge, no previous study has investigated GATA-3 expression in canine UC.

The Ki67 is an example of a biomarker widely used in both human and veterinary oncology to establish prognostic estimates, either alone or in association with other markers (6, 18). Protein Ki67 expression is an indicator of cell proliferation within a population of cells (19). This expression has already been evaluated in canine cancers, such as lymphoma, melanoma, mast cell tumors, and mammary tumors (18, 20–22). Regarding bladder UC, several studies in humans have shown that Ki67 is an important factor to consider in the prognosis and tumor behavior related to disease progression and histological grade (6, 23–27). In veterinary medicine, to the best of our knowledge, only one study has investigated the expression of Ki67 in canine bladder UC, with no statistically significant correlation between this marker and clinicopathological findings (28), showing the importance of studies related to Ki67 for this tumor type, and associating it with histopathological features.

Thus, this study aimed to evaluate Caveolin-1, GATA-3, and Ki67 expression in canine UC samples and correlate this with pathological variables.

## Materials and methods

### Ethic statement

This study was approved by the Institutional Ethics Committee on the Use of Animals in Research from São Paulo State University- UNESP (Protocol 50/2020). All owners signed an informed consent form, allowing the use of the patient's samples in the research.

### Study design

This retrospective non-randomized study included 30 canine UC samples conferred to the VetPat Laboratory (21 cases) and the University of Milan (9 cases) between January 2000 and June 2019. The inclusion criteria were as follows: (i) patients who underwent tissue biopsy or surgical procedures to acquire tissue samples, (ii) availability of tissue samples in paraffin blocks for immunohistochemistry, and (iii) clinicopathological information. Clinical data were obtained from the records of each animal and the following histopathological parameters were evaluated: histological type and grade, muscle invasion, lymphatic invasion, and mitotic index. Samples whose material was insufficient to determine the histological type (infiltrating or not infiltrating) or that presented negative internal controls for the markers were excluded.

### Histological analysis

Hematoxylin and eosin staining was performed for tumor classification. The histological subtypes were obtained by three evaluators (VMG, VG, and RL-A) according to the criteria proposed by the World Health Organization (23, 29), and histological grading was performed according to Valli et al. (30). In addition, the presence or absence of tumor infiltration in the muscle layer of bladder and the presence or absence of lymphatic invasion was reported. Mitotic count was obtained by counting the total number of cells undergoing mitoses with high mitotic activity, totaling an area of 2.37 mm^2^ (400× magnification), according to Romansik et al. (31).

### Immunohistochemical analysis

#### Ki67 immunoexpression

From each formalin-fixed and paraffin-embedded sample, 5 μm-thick sections were cut. Histological sections were deparaffinized and rehydrated using graded alcohols, and endogenous peroxidase activity was blocked for 30 min in a 0.3% H_2_O_2_ methanol solution. Heat-induced antigen retrieval was performed using a pressure cooker for 20 min in a citrate buffer (pH 6.0). After washing in Tris-buffer, protein blocking was performed using normal horse serum for 30 min at 25°C. Sections were then incubated with a mouse anti-human Ki-67 primary antibody (MIB-1; Dako, CA, USA) diluted 1:600 in Tris buffer for 18 h at 4°C. After rinsing sections in Tris buffer, slides were incubated with a biotinylated horse anti-mouse secondary antibody (Vector Laboratories, CA, USA) for 30 min at 25°C. Immunohistochemical signals were detected using an avidin-biotin system (Vector Laboratories, CA, USA) and 3-amino-9-ethylcarbazole (AEC) substrate-chromogen kit (Vector Laboratories, CA, USA). Sections were counterstained with Harris hematoxylin and mounted using an aqueous mounting medium (Aquatex, Sigma-Aldrich, MO, USA). The Ki-67 value was expressed as the percentage of positively stained cells, calculated by counting 1,000 cells per section (400× magnification).

#### GATA-3 and Caveolin-1 immunoexpression

Samples were processed similarly to the Ki67 marker and were submitted to the antigenic retrieval procedure: Caveolin-1 in citric acid solution (pH 6.0) in a pressure cooker, and GATA-3 in a high pH buffer solution (EnVision Flex, High pH, Dako, CA, USA) in water bath at 98°C for 30 min. Endogenous peroxidase was blocked with 0.5% H_2_O_2_ methanol solution for 20 min; and later with diluted powdered milk (3 g in 100 ml) at 25°C for 1 h. The slides were incubated in a humid chamber with the primary antibodies, including anti-human monoclonal mouse GATA-3 (1:100, L50-823, Biocare Medical, CA, USA), and CAV1 anti-human polyclonal (1:1,000, AVARP09019_T100, Aviva Systems Biology, CA, USA). After washing with Tris-buffer, the sections were incubated with the secondary antibody (EnVision Flex SM802, Dako, CA, USA) at 37°C for 1 h, and the immunohistochemical signal was detected using a diaminobenzidine solution (DAB Chromogen Kit, Dako, CA, USA). Counterstaining was performed with Harris hematoxylin.

The expression of Caveolin-1 was analyzed semi-quantitatively using the criteria intensity of labeling from 1 to 3 and tumor staining distribution from 1 to 4 (1 = 1–25%, 2 = 26–50%, 3 = 51–75%, 4 > 75%). The intensity and distribution values were then multiplied, resulting in a value from 1 to 12, which was representative of the expression of this marker. The expression of GATA-3 was analyzed quantitatively, and its value was expressed as the percentage of positively stained cells calculated by counting 1,000 cells per section (400 × magnification).

### Statistical analysis

The pathological variables were tabulated, and prevalence was calculated as the percentage of occurrence among all cases. In this case, the results were presented as means and standard deviations. The biomarker values and histopathological information were associated in univariate analysis using the Student's *t*-test or Mann-Whitney test when the pathological variable was composed of two groups, or the Kruskal-Wallis test when the variable was composed of three groups. Multivariate analysis was performed to correlate all variables using Spearman's correlation test. The correlation coefficients (r) were interpreted according to Pett, Lackey, and Sullivan (32), dividing the classifications into weak (0–0.29), low (0.3–0.49), moderate (0.5–0.69), strong (0.7–0.89), or very strong (0.9–1.0); whether they are positive or negative. No weak correlation was observed.

The muscular invasion variable was not considered in the tests because of the small number of patients with this information. The software used was GraphPad Prism v 8.0.1 (GraphPad Software Inc., La Jolla, CA, USA).

## Results

### Clinicopathological and immunohistochemical data

Thirty UC samples were obtained, and clinicopathological information is presented in Table 1. All samples were classified for the histological type, divided into papillary and infiltrating (60%) and non-papillary and infiltrating (40%), with the invasive characteristics being observed in all cases. The histological grade II was observed in 63.33% of the cases, followed by grade III (30.01%), and grade I (6.66%) (Table 1). Of the 30 bladder tumors, 20 presented with all layers for histological evaluation of muscle invasion. Of these complete samples, 50% already had some infiltration of the bladder muscle layer. In addition, all 30 tumor samples were evaluated for lymphatic invasion, and 53.33% demonstrated the presence of invasion of lymphatic vessels (Table 1).

**Table 1:** Clinicopathological and immunohistochemical data.

**Case**	**Sex**	**Age (years)**	**Histologic classification** **(WHO, 2004)**	**Histologic grade (30)**	**Sample with all layers**	**Muscular invasion**	**Lymphatic invasion**	**Mitotic count (mc)**	**Ki67**	**Caveolin-1**	**Gata-3 %**	**Survival (days)**
1	Male	11	TCC Papillary and infiltrating	II	Yes	Yes	PRESENT AND EXTENSIVE	2	6.05%	(3 × 3) = 9	51.70	90
2	Female	15	TCC Papillary and infiltrating	II	No	NI	ABSENT	39	40.20%	(4 × 3) = 12	35.75	NI
3	Female	14	TCC Papillary and infiltrating	II	No	NI	ABSENT	4	25.09%	(2 × 3) = 6	70.24	101
4	Female	5	TCC Papillary and infiltrating	II	Yes	No	PRESENT	24	45.91%	(4 × 3) = 12	91.64	NI
5	Female	10	TCC Non-papillary and infiltrating	III	Yes	Yes	PRESENT AND EXTENSIVE	27	30.80%	(2 × 1) = 2	87.04	130
6	Female	7	TCC Papillary and infiltrating	II	No	NI	ABSENT	28	42.72%	(1 × 1) = 1	23.32	NI
7	Female	15	TCC Papillary and infiltrating	III	No	NI	PRESENT	17	20.33%	(4 × 3) = 12	66.94	NI
8	Female	8	TCC Non-papillary and infiltrating	II	Yes	No	ABSENT	120	76.34%	(2 × 2) = 4	90.96	NI
9	Female	14	TCC Non-papillary and infiltrating	II	Yes	Yes	ABSENT	7	3.48%	(2 × 2) = 4	88.18	NI
10	Female	7	TCC Papillary and infiltrating	II	Yes	No	PRESENT	22	33.33%	(3 × 2) = 6	70.28	NI
11	Female	15	TCC Papillary and infiltrating	II	No	NI	ABSENT	81	27.98%	(1 × 2) = 2	87.21	NI
12	Female	10	TCC Papillary and infiltrating	II	No	NI	PRESENT	12	35.66%	(3 × 2) = 6	75.28	NI
13	Male	11	TCC Non-papillary and infiltrating	II	Yes	Yes	PRESENT	7	34.40%	(4 × 3) = 12	77.61	60
14	Female	12	TCC Non-papillary and infiltrating	II	Yes	No	PRESENT AND EXTENSIVE	78	54.97%	(1 × 2) = 2	87.37	NI
15	Female	13	TCC Papillary and infiltrating	II	No	NI	PRESENT	31	10.33%	(1 × 2) = 2	80.51	NI
16	Male	10	TCC Non-papillary and infiltrating	III	Yes	Yes	PRESENT AND EXTENSIVE	66	14.25%	(2 × 2) = 4	71.04	180
17	Female	10	TCC Non-papillary and infiltrating	III	No	NI	PRESENT	51	16.27%	(1 × 2) = 2	88.30	NI
18	Female	8	TCC Non-papillary and infiltrating	III	Yes	Yes	PRESENT AND EXTENSIVE	12	33.38%	(3 × 2) = 6	89.04	NI
19	Female	12	TCC Non-papillary and infiltrating	III	Yes	No	PRESENT	21	24.48%	(4 × 3) = 12	66.86	NI
20	Female	13	TCC Papillary and infiltrating	II	Yes	No	ABSENT	22	29.53%	(1 × 2) = 2	58.42	201
21	Male	15	TCC Non-papillary and infiltrating	III	Yes	No	PRESENT	22	11.69%	(3 × 2) = 6	70.35	NI
22	Male	10	TCC Non-papillary and infiltrating	III	Yes	Yes	ABSENT	28	25.36%	(3 × 2) = 6	61.94	NI
23	Female	13	TCC Papillary and infiltrating	II	Yes	No	ABSENT	30	6.84%	(2 × 1) = 2	78.09	NI
24	Male	11	TCC Papillary and infiltrating	II	Yes	Yes	PRESENT	17	10.19%	(1 × 1) = 1	78.90	NI
25	Male	8	TCC Papillary and infiltrating	II	Yes	Yes	ABSENT	NI	8.24%	(4 × 3) = 12	78.88	NI
26	Female	12	TCC Papillary and infiltrating	II	No	NI	ABSENT	32	17.72%	(3 × 3) = 9	84.88	NI
27	Female	15	TCC Papillary and infiltrating	II	Yes	No	ABSENT	28	4.78%	(4 × 3) = 12	85.14	NI
28	Male	NI	TCC Papillary and infiltrating	I	No	NI	ABSENT	NI	4.31%	(2 × 3) = 6	56.13	NI
29	Male	15	TCC Non-papillary and infiltrating	III	Yes	Yes	PRESENT	27	12.75%	(3 × 3) = 9	79.43	NI
30	Male	10	TCC Papillary and infiltrating	I	Yes	No	ABSENT	36	16.84%	(3 × 2) = 6	89.55	NI

NI, No information.

For histological evaluation of cell proliferation in canine UC, mitotic count (MC) was considered. A total of 28 samples allowed the determination of MC through the visualization of the necessary tumoral area (Table 1), and the MC was 31.82 ± 26.26 (mean ± standard deviation).

**Figure 1:**
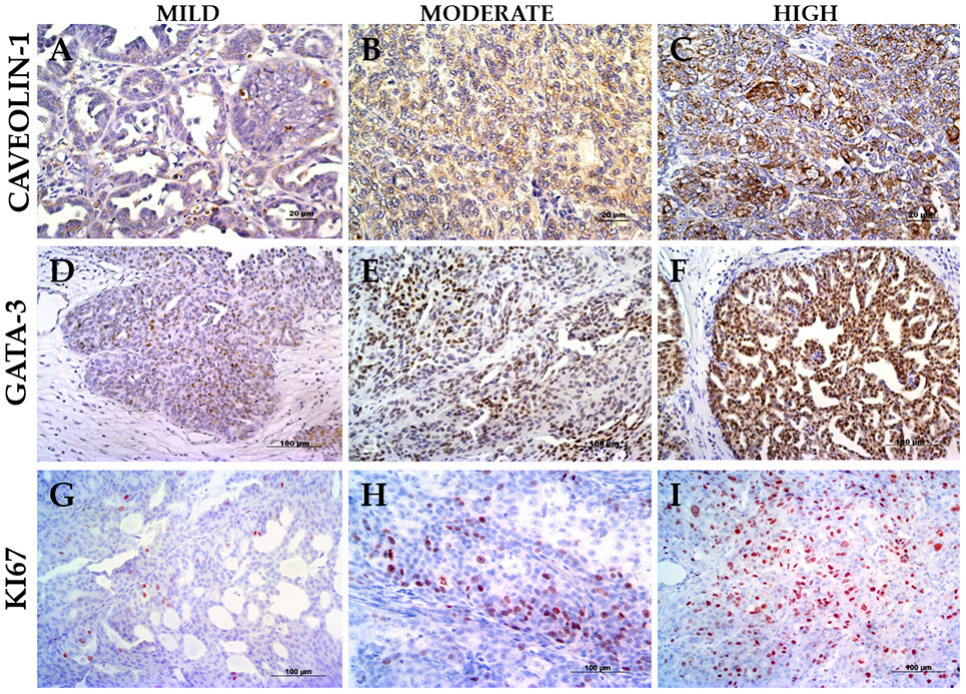
Immunostaining panel. **(A)** UC papillary and infiltrating with mild Caveolin-1 (case 6), 400× magnification. **(B)** UC papillary and infiltrating with intermediate Caveolin-1 (case 30), 400× magnification. **(C)** UC papillary and infiltrating with high Caveolin-1 (case 7), 400× magnification. **(D)** UC papillary and infiltrating with mild GATA-3 (case 2), 200× magnification. **(E)** UC non-papillary and infiltrating with intermediate GATA-3 (case 22), 200× magnification. **(F)** UC papillary and infiltrating with high GATA-3 (case 30), 200× magnification. **(G)** UC papillary and infiltrating with mild Ki67 (case 15), 200× magnification. **(H)** UC papillary and infiltrating with intermediate Ki67 (case 26), 200× magnification. **(I)** UC non-papillary and infiltrating with high Ki67 (case 14), 200× magnification.

As per immunohistochemistry analysis, the immunostaining values of Ki67, GATA-3, and Caveolin-1 were 24.14 ± 16.88, 71.40 ± 16.38, and 6.23 ± 3.95% (median ± standard deviation), respectively (Figure 1 and Table 1). The quantitative expression of GATA-3 was >75% in 60% of the cases, and the semi-quantitative expression of Caveolin-1 was ≥6 in 60% of the canine UC cases. Examples of the different expressions of Ki67, GATA-3, and Caveolin-1 are illustrated in Figure 1. Survival data were retrieved from only six of the total dogs, and the median overall survival was 127 days after diagnosis.

### Association between all clinicopathological and immunohistochemistry variables

**Figure 2:**
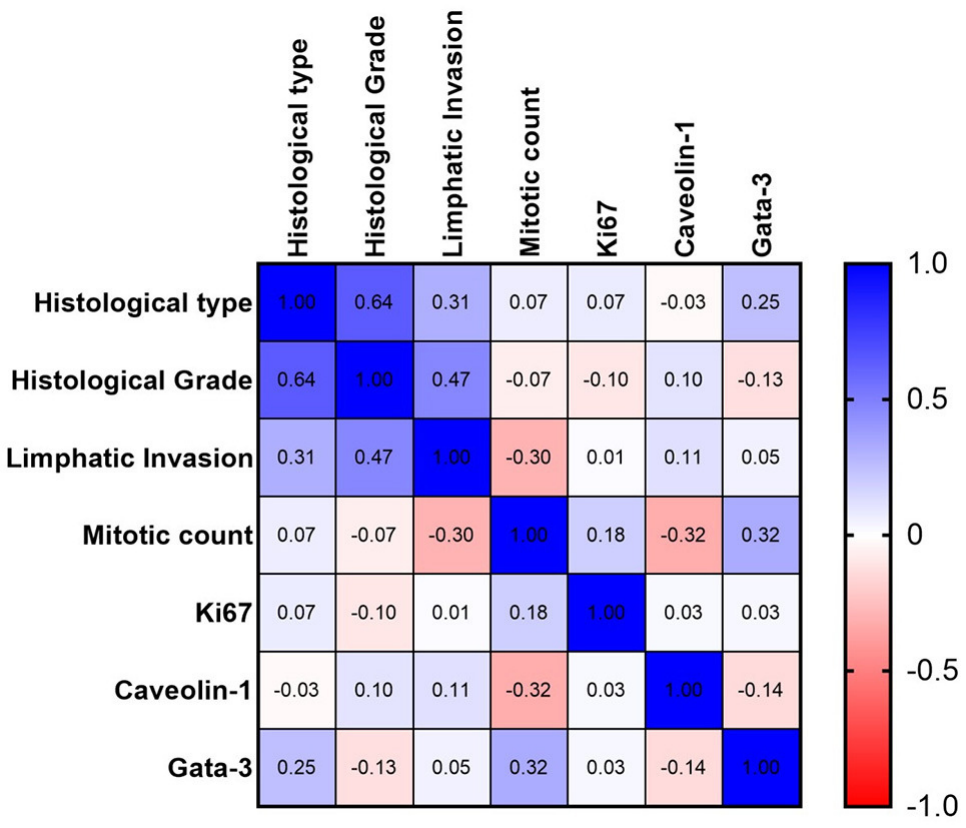
Spearman's graph of multiple correlations with the respective correlation coefficients. Blue color indicate a positive correlation and red color a negative correlation. The intensity of the color is related with a stronger correlation. A strong correlation between histological grade and histological type and a moderate negative correlation between mitotic count and Caveolin-1 expression are observed.

In the multivariate analysis of multiple correlations, some interesting results were obtained (Figure 2). GATA-3 expression positively correlated with MC (weak correlation, *r* = 0.32). Thus, samples with higher MC also showed high GATA-3 expression. Caveolin-1 was negatively correlated with MC (weak correlation, *r* = −0.32), with a higher MC corresponding to low Caveolin-1 expression. The ki67 was not significantly correlated with any of the variables (Figure 2 and Table 2).

**Table 2:** *P* values for the univariate analysis.

***X***	**Mitotic count**	**Ki67**	**Caveolin-1**	**GATA-3**
Histological type	0.7401	0.4648	0.6616	0.1461
Histological grade	0.6489	0.3742	0.8892	0.9916
Lymphatic invasion	0.1235	0.5249	0.7857	0.6084
Mitotic count	–	0.3466	0.099	0.0917
Ki67	0.3466	–	0.8484	0.6595
Caveolin-1	0.099	0.8484	–	0.4415
GATA-3	0.0917	0.6595	0.4415	–

MC and immunohistochemical markers values were associated with each other and with the histopathological variables, without statistically significant results.

## Discussion

Canine UC is known for its increased invasiveness at the time of diagnosis and is an important model for muscle-invasive diseases in humans (1, 4). However, despite the difficulties associated with the treatment of this tumor type in dogs, most studies related to biomarkers for bladder UC in veterinary medicine analyze isolated molecules without assessing their prognostic value or association with other variables (7). For this reason, we investigated the expression of Caveolin-1 and GATA-3 in canine UC to assess their expression patterns and associate them with clinicopathological findings. In our opinion, this assessment is very important because we lack prognostic and predictive markers for canine UC. Although tumor biopsy is essential for investigating these markers, UC diagnosis is usually performed using cytological or molecular (BRAF assessment) tools in veterinary medicine. Therefore, studies using tumor biopsy are pivotal for proposing new markers and stimulating biopsy procedures in UC-affected dogs.

Of the 30 cases studied, all were histologically classified as infiltrating, with 50% of 20 samples showing muscle invasion and 53.33% of the total showing invasion of lymphatic vessels. These results reinforce the invasive characteristics of most cases of bladder tumors in dogs and highlight the importance of studying new predictive biomarkers. This result also corroborates with the cases found in the clinical routine, in which clinicians and surgeons found more dogs with advanced disease, surgery not being the therapeutic option. In this study, muscle invasiveness was not assessed in 10 of the 30 cases because the biopsy samples did not present all bladder layers. Therefore, it is important to reinforce the necessity for surgeons to collect representative samples for inclusion in this analysis. Interestingly, muscle invasion was found in 10 of the 20 cases in which all layers were present.

In a previous study, our research group identified an association between lymphatic invasion and poor prognosis in canine UC (33). Because some of the samples used were also used in this study, we did not associate survival with lymphatic invasion to avoid data duplication. However, since we increased the number of samples and assessed some immunohistochemical markers, we performed a new analysis and identified different associations. Tumors with higher histological grades and more aggressive histological subtypes were correlated with lymphatic invasion. Thus, these results complement our previous findings and reinforce the importance of collecting biopsies for canine UC diagnosis and assessment of histological criteria such as lymphatic invasion.

The role of Ki67 in tumor behavior has been extensively studied in several cancers in veterinary medicine, including mast cell tumors (18). Similarly, several studies have evaluated the prognostic value of Ki67 for human UC. Chirife et al. (24) reported Ki67 as a predictor of bladder tumor progression in patients undergoing initial transurethral resection, and a meta-analysis by He et al. (6) found a statistically significant correlation between the overexpression of Ki67 and shorter progression-free survival. The present study did not correlate the Ki67 values with survival data. In addition, due to the small number of reported cases of overall survival and the retrospective nature of the study, variables such as progression-free survival were not evaluated.

The association of Ki67 values with other histological variables is also important for a better understanding of the tumor biology of bladder UC in both humans and dogs. Gönül et al. (23) observed an association between this biomarker and tumor grade in humans; however, the same association was not found in the present study. Although in human medicine there are many articles investigating the predictive value of Ki67 for bladder UC, to the best of our knowledge, Hanazono et al. (28) were the first authors to investigate the correlation between Ki67 and survival time, with the present study the second one correlating Ki67 with the other two biomarkers and histological features. Therefore, no significant statistical correlation was found between Ki67 and any other variable, stimulating the execution of more studies with a larger number of animals and more follow-up information.

In addition to Ki67, other markers have been studied in terms of their significance in tumor behavior in human UC, such as GATA-3 and Caveolin-1 (9, 10, 15–17). However, no veterinary studies have investigated these markers for the same disease in dogs, except for a comparative study that used dogs as a model of human disease, subclassifying canine bladder carcinomas into molecular subtypes (11). Although these markers were only observed in human disease to date, Naik et al. (16) observed a lower positivity of this marker in high-grade UC when compared to low-grade UC, in tumors with muscle invasion when compared to those with invasion only in the lamina propria, and in non-papillary tumors when compared to papillary histological types. Leivo et al. (34) did not demonstrate the prognostic significance of GATA-3 for UC, and Miyamoto et al. (15) showed that while this biomarker was less expressed in low-grade and muscle-invasive tumors, its strong immunostaining proved to be an independent predictor of poor prognosis. These results highlight the controversial and poorly understood biological role of GATA-3 in UC.

In contrast to most of the correlations mentioned above for this disease in humans, the present study found a positive and low correlation only between GATA-3 and MC, indicating that tumors with higher GATA-3 expression tend to have higher MC. Considering that high mitotic activity may predict a greater chance of tumor recurrence in human UC (35) and, consequently, more aggressive tumor behavior, this result suggests that higher GATA-3 positivity can indicate a worse prognosis for bladder cancer in dogs. However, it should be noted that all samples used already had an invasive character in the lamina propria or muscle layer, making this sample set homogeneous for this item, which possibly influenced the tangentially higher mitotic index of these cases. The invasive character of the tumor samples may also have influenced the non-correlation of Ki67 values with the other variables.

Similar to GATA-3, Caveolin-1 expression correlated with MC in this study. However, the correlation was low and negative, indicating that higher expression values of this marker tended to be accompanied by lower MC values. The results found in humans are still unclear; Fong et al. (9) reported a positive correlation between Caveolin-1 expression and tumor histological grade, and D'Andrea et al. (10) showed a positive association not only between Caveolin-1 values and tumor grade, but also with more advanced stages of the disease. In contrast, this previous study also showed that higher Caveolin-1 levels were associated with lower disease recurrence. The present study results are contrary to most results in humans, as they indicate that high expression values of Caveolin-1 can indicate a favorable tumor biological behavior, as they were inversely correlated with MC. Therefore, the same bias of high histological invasiveness of tumor samples must be considered. Furthermore, for GATA-3 and Caveolin-1, the correlations found were classified as low, indicating the need for further studies with the same biomarkers.

Concerning histological variables such as MC, histological type, histological grade, and lymphatic invasion, a low positive correlation was found between lymphatic invasion and histological type, as well as between lymphatic invasion and the histological grade by Valli (1995), suggesting that non-papillary tumors, as well as high histological grade tumors, are more likely to present lymphatic vessel invasion. Considering that the presence of lymphatic invasion of tumor cells is an important prognostic factor related to the survival and recurrence of this disease in the upper and lower urinary tract (36–38), it is suggested that the type and histological grade may also have a prognostic predictive value and should always be carefully evaluated and considered when establishing treatment for canine bladder UC cases. More studies should be carried out to confirm and investigate the predictive potential of this histological variable and the biomarkers GATA-3 and Caveolin-1 for canine bladder UC, preferably correlating them with overall survival.

GATA-3 and Caveolin-1 have been demonstrated as potential biomarkers of the biological behavior of canine bladder carcinoma, correlating with MC. The histopathological type and grade were also considered important factors to be carefully evaluated at the time of diagnosis as potential predictors of this tumor behavior, correlating with lymphatic invasion. More studies are necessary to further investigate the significance of Ki67, GATA-3, and Caveolin-1 expression in canine bladder UC, preferentially associating them with overall survival.

## Data availability statement

The original contributions presented in the study are included in the article/supplementary material, further inquiries can be directed to the corresponding authors.

## Ethics statement

The animal study was reviewed and approved by this study was previously approved by the Institutional Ethics Committee on the use of Animal in Research from São Paulo State University- UNESP (Protocol 50/2020).

## Author contributions

VMG, CF-A, and JG: conceptualization. VMG, CF-A, VG, RL-A, and JG: methodology. VMG, CP, and CF-A: software. VMG, CF-A, VG, and RL-A: validation. VMG and CF-A: formal analysis. VMG, CP, EB, FR, CF-A, RL-A, and VG: investigation. CF-A, RL-A, and RT: resources. VG, CP, CF-A, and RL-A: data curation. VMG: writing—original draft preparation. VMG, CF-A, CP, RL-A, and VG: writing—review and editing. VMG, CF-A, RL-A, CP, and VG: visualization. CF-A: supervision. CF-A, JG, and VG: project administration. All authors have read and agreed to the published version of the manuscript.

## Funding

This research was founded in part by São Paulo Research Foundation (FAPESP), grant number # 2019/24079-1 and by the National Council for Scientific and Technological Development (CNPq), grant numbers # 422139/2018-1 and #302977/2021-0.

## Conflict of interest

The authors declare that the research was conducted in the absence of any commercial or financial relationships that could be construed as a potential conflict of interest.

## Publisher's note

All claims expressed in this article are solely those of the authors and do not necessarily represent those of their affiliated organizations, or those of the publisher, the editors and the reviewers. Any product that may be evaluated in this article, or claim that may be made by its manufacturer, is not guaranteed or endorsed by the publisher.

## References

[1] Knapp DW Dhawan D Ramos-Vara JA Ratliff TL Cresswell GM Utturkar S. Naturally-occurring invasive urothelial carcinoma in dogs, a unique model to drive advances in managing muscle invasive bladder cancer in humans. Front Oncol. (2019) 9:1493. 10.3389/fonc.2019.01493 32039002 PMC6985458

[2] Antoni S Ferlay J Soerjomataram I Znaor A Jemal A Bray F. Bladder cancer incidence and mortality: a global overview and recent trends. Eur Urol. (2017) 71:96–108. 10.1016/j.eururo.2016.06.010 27370177

[3] Siegel RL Miller KD Jemal A. Cancer statistics, 2019. CA Cancer J Clin. (2019) 69:7–34. 10.3322/caac.21551 30620402

[4] Knapp DW Ramos-Vara JA Moore GE Dhawan D Bonney PL Young KE. Urinary bladder cancer in dogs, a naturally occurring model for cancer biology and drug development. ILAR J. (2014) 55:100–18. 10.1093/ilar/ilu018 24936033

[5] Ro JY Staerkel GA Ayala AG. Cytologic and histologic features of superficial bladder cancer. Urol Clin North Am. (1992) 19:435–53. 10.1016/S0094-0143(21)00412-2 1636229

[6] He Y Wang N Zhou X Wang J Ding Z Chen X. Prognostic value of ki67 in BCG-treated non-muscle invasive bladder cancer: a meta-analysis and systematic review. BMJ Open. (2018) 8:e019635. 10.1136/bmjopen-2017-019635 29666128 PMC5905754

[7] Gambim VV Laufer-Amorim R Fonseca-Alves RH Grieco V Fonseca-Alves CE. A comparative meta-analysis and in silico analysis of differentially expressed genes and proteins in canine and human bladder cancer. Front Vet Sci. (2020) 7:558978. 10.3389/fvets.2020.558978 33304937 PMC7701042

[8] Hanazono K Fukumoto S Kawamura Y Endo Y Kadosawa T Iwano H. Epidermal growth factor receptor expression in canine transitional cell carcinoma. J Vet Med Sci. (2015) 77:1–6. 10.1292/jvms.14-0032 25223345 PMC4349531

[9] Fong A Garcia E Gwynn L Lisanti MP Fazzari MJ Li M. Expression of caveolin-1 and caveolin-2 in urothelial carcinoma of the urinary bladder correlates with tumor grade and squamous differentiation. Am J Clin Pathol. (2003) 120:93–100. 10.1309/292NHAYNWAVREJ37 12866378

[10] D'Andrea D Moschini M Foerster B Abufaraj M Margulis V Karam J. Caveolin-1 expression in upper tract urothelial carcinoma. Eur Urol Focus. (2019) 5:97–103. 10.1016/j.euf.2017.06.011 28753840

[11] Williams TM Lisanti MP. The caveolin proteins. Genome Biol. (2004) 5:214. 10.1186/gb-2004-5-3-214 15003112 PMC395759

[12] Deb M Sengupta D Kar S Rath SK Parbin S Shilpi A. Elucidation of caveolin 1 both as a tumor suppressor and metastasis promoter in light of epigenetic modulators. Tumour Biol. (2014) 35:12031–47. 10.1007/s13277-014-2502-z 25192721

[13] Miettinen M McCue PA Sarlomo-Rikala M Rys J Czapiewski P Wazny K. GATA3: a multispecific but potentially useful marker in surgical pathology: a systematic analysis of 2500 epithelial and nonepithelial tumors. Am J Surg Pathol. (2014) 38:13–22. 10.1097/PAS.0b013e3182a0218f 24145643 PMC3991431

[14] Patient RK McGhee JD. The GATA family (vertebrates and invertebrates). Curr Opin Genet Dev. (2002) 12:416–22. 10.1016/S0959-437X(02)00319-2 12100886

[15] Miyamoto H Izumi K Yao JL Li Y Yang Q McMahon LA. GATA binding protein 3 is down-regulated in bladder cancer yet strong expression is an independent predictor of poor prognosis in invasive tumor. Hum Pathol. (2012) 43:2033–40. 10.1016/j.humpath.2012.02.011 22607700

[16] Naik M Rao BV Fonseca D Murthy SS Giridhar A Sharma R. GATA-3 Expression in all grades and different variants of primary and metastatic urothelial carcinoma. Indian J Surg Oncol. (2021) 12:72–8. 10.1007/s13193-019-01026-0 33994731 PMC8119539

[17] Liang Y Heitzman J Kamat AM Dinney CP Czerniak B Guo CC. Differential expression of GATA-3 in urothelial carcinoma variants. Hum Pathol. (2014) 45:1466–72. 10.1016/j.humpath.2014.02.023 24745616

[18] Fonseca-Alves CE Bento DD Torres-Neto R Werner J Kitchell B Laufer-Amorim R. Ki67/KIT double immunohistochemical staining in cutaneous mast cell tumors from Boxer dogs. Res Vet Sci. (2015) 102:122–6. 10.1016/j.rvsc.2015.08.007 26412531

[19] Abadie JJ Amardeilh MA Delverdier ME. Immunohistochemical detection of proliferating cell nuclear antigen and Ki-67 in mast cell tumors from dogs. Am Vet Med Assoc. (1999) 215:1629–34. 14567425

[20] Sierra Matiz OR Santilli J Anai LA Da Silva MCL Sueiro FA Sequeira JL. Prognostic significance of Ki67 and its correlation with mitotic index in dogs with diffuse large B-cell lymphoma treated with 19-week CHOP-based protocol. J Vet Diagn Invest. (2018) 30:263–7. 10.1177/1040638717743280 29192554 PMC6505881

[21] Bergin IL Smedley RC Esplin DG Spangler WL Kiupel M. Prognostic evaluation of Ki67 threshold value in canine oral melanoma. Vet Pathol. (2011) 48:41–53. 10.1177/0300985810388947 21123859

[22] Kaszak I Ruszczak A Kanafa S Kacprzak K Król M Jurka P. Current biomarkers of canine mammary tumors. Acta Vet Scand. (2018) 60:66. 10.1186/s13028-018-0417-1 30373614 PMC6206704

[23] Gönül II Akyürek N Dursun A Küpeli B. Relationship of Ki67, TP53, MDM-2 and BCL-2 expressions with WHO 1973 and WHO/ISUP grades, tumor category and overall patient survival in urothelial tumors of the bladder. Pathol Res Pract. (2008) 204:707–17. 10.1016/j.prp.2008.03.011 18572327

[24] Chirife AM Villasante N Rojas Bilbao É Casas G. Individual patient risk of progression of urinary bladder papillary tumors estimated from biomarkers at initial transurethral resection of bladder tumor. J Cancer Res Clin Oncol. (2019) 145:1709–18. 10.1007/s00432-019-02923-1 31030273 PMC11810434

[25] Geelvink M Babmorad A Maurer A Stöhr R Grimm T Bach C. Diagnostic and prognostic implications of FGFR3. Int J Mol Sci. (2018) 19:2548. 10.3390/ijms19092548 30154342 PMC6163244

[26] Wang L Feng C Ding G Ding Q Zhou Z Jiang H. Ki67 and TP53 expressions predict recurrence of non-muscle-invasive bladder cancer. Tumour Biol. (2014) 35:2989–95. 10.1007/s13277-013-1384-9 24241960

[27] Thakur B Kishore S Dutta K Kaushik S Bhardwaj A. Role of p53 and Ki-67 immunomarkers in carcinoma of urinary bladder. Indian J Pathol Microbiol. (2017) 60:505–9. 10.4103/IJPM.IJPM_246_17 29323062

[28] Hanazono K Nishimori T Fukumoto S Kawamura Y Endo Y Kadosawa T. Immunohistochemical expression of p63, Ki67 and β-catenin in canine transitional cell carcinoma and polypoid cystitis of the urinary bladder. Vet Comp Oncol. (2016) 14:263–9. 10.1111/vco.12095 24758385

[29] Meuten DJ Everitt J Inskeep W Jacobs RM Peleteiro M Thompson KG. Histological Classification of Tumors of the Urinary System o Domestic Animals. Washington, DC: World Health Organization (2004).

[30] Valli VE Norris A Jacobs RM Laing E Withrow S Macy D. Pathology of canine bladder and urethral cancer and correlation with tumour progression and survival. J Comp Pathol. (1995) 113:113–30. 10.1016/S0021-9975(05)80027-1 8543669

[31] Romansik EM Reilly CM Kass PH Moore PF London CA. Mitotic index is predictive for survival for canine cutaneous mast cell tumors. Vet Pathol. (2007) 44:335–41. 10.1354/vp.44-3-335 17491075

[32] Pett MA Lackey NR Sullivan JJ. Making Sense of Factor Analysis: The Use of Factor Analysis for Instrument Development in Health Care Research. Thousand Oaks, CA: Sage. (2003). 10.4135/9781412984898

[33] Govoni VM Pigoli C Sueiro FAR Zuliani F da Silva TO Quitzan JG. Lymphatic invasion is a significant indicator of poor patient outcome in canine bladder urothelial carcinoma. Open Vet J. (2021) 11:535–43. 10.5455/OVJ.2021.v11.i4.3 35070848 PMC8770177

[34] Leivo MZ Elson PJ Tacha DE Delahunt B Hansel DE. A combination of p40, GATA-3 and uroplakin II shows utility in the diagnosis and prognosis of muscle-invasive urothelial carcinoma. Pathology. (2016) 48:543–9. 10.1016/j.pathol.2016.05.008 27594510

[35] Zaleski M Gogoj A Walter V Raman JD Kaag M Merrill SB. Mitotic activity in noninvasive papillary urothelial carcinoma: its value in predicting tumor recurrence and comparison with the contemporary 2-tier grading system. Hum Pathol. (2019) 84:275–82. 10.1016/j.humpath.2018.10.008 30359638

[36] Brunocilla E Pernetti R Martorana G. The prognostic role of lymphovascular invasion in urothelial-cell carcinoma of upper and lower urinary tract. Anticancer Res. (2011) 31:3503–6. 21965769

[37] Danzig MR Mallin K McKiernan JM Stadler WM Sridhar SS Morgan TM. Prognostic importance of lymphovascular invasion in urothelial carcinoma of the renal pelvis. Cancer. (2018) 124:2507–14. 10.1002/cncr.31372 29624636 PMC8248269

[38] Mathieu R Lucca I Rouprêt M Briganti A Shariat SF. The prognostic role of lymphovascular invasion in urothelial carcinoma of the bladder. Nat Rev Urol. (2016) 13:471–9. 10.1038/nrurol.2016.126 27431340

